# ELSA-Brasil: a 4-year incidence of hearing loss in adults with and without hypertension

**DOI:** 10.11606/s1518-8787.2022056003796

**Published:** 2022-04-11

**Authors:** Fernanda Yasmin Odila Maestri Miguel Padilha, Nágila Soares Xavier Oenning, Itamar de Souza Santos, Camila Maia Rabelo, Renata Rodrigues Moreira, Isabela M. Bensenor, Paulo A. Lotufo, Alessandra Giannella Samelli

**Affiliations:** I Universidade de São Paulo Faculdade de Medicina Departamento de Fisioterapia, Fonoaudiologia e Terapia Ocupacional São Paulo SP Brasil Universidade de São Paulo. Faculdade de Medicina. Departamento de Fisioterapia, Fonoaudiologia e Terapia Ocupacional. São Paulo, SP, Brasil; II Petrobrás Florianópolis SC Brasil Petrobrás. Florianópolis, SC, Brasil; III Pontificia Universidad Católica del Ecuador Quito Pichincha Ecuador Pontificia Universidad Católica del Ecuador. Quito, Pichincha, Ecuador; IV Universidade de São Paulo Hospital Universitário Centro de Pesquisa Clínica e Epidemiológica São Paulo SP Brasil Universidade de São Paulo. Hospital Universitário. Centro de Pesquisa Clínica e Epidemiológica, São Paulo, SP, Brasil; V Universidade de São Paulo Faculdade de Medicina Departamento de Clínica Médica São Paulo SP Brasil Universidade de São Paulo. Faculdade de Medicina. Departamento de Clínica Médica. São Paulo, SP, Brasil; VI Universidade de São Paulo Hospital Universitário São Paulo SP Brasil Universidade de São Paulo. Hospital Universitário. Serviço de Audiologia. São Paulo, SP, Brasil

**Keywords:** Adult, Hearing Loss, Hypertension, Epidemiologic Factors, Longitudinal Studies

## Abstract

**OBJECTIVE:**

To compare the incidence of hearing loss among adults stratified by the occurrence of hypertension, and to investigate the association between hypertension and hearing loss.

**METHODS:**

Longitudinal observational study, part of the *Estudo Longitudinal da Saúde do Adulto* (ELSA-Brasil, Longitudinal Study on Adult’s Health). Data from the first and second waves were analyzed, including information from audiological assessment and general health of the subjects. As outcome, we considered the presence of hearing loss (hearing thresholds above 25 dBHL at frequencies from 500 Hz to 8 kHz) and, as exposure variable, hypertension (report of medical diagnosis of hypertension; and/or use of drugs to treat hypertension; and/or pressure systolic blood pressure ≥ 140 mmHg; or diastolic blood pressure ≥ 90 mmHg). As covariables for adjustment were considered: sex, age, education, race / ethnicity, income, smoking, diabetes, and occupational exposure to noise. Poisson regression analysis was conducted, estimating the crude and adjusted relative risks, with 95% confidence intervals, in order to assess the factors associated with hearing loss.

**RESULTS:**

In crude analyses, the incidence of hearing loss was higher for subjects with hypertension (9.7% versus 5.4%). The crude relative risks for hearing loss was almost double (1.93; 95%CI: 1.10–3.39) for subjects with hypertension in the right ear. In the adjusted analyses, the relative risks was not significant for the hypertension variable (1.42; 95%CI: 0.75–2.67). Being 60 years or older (RR: 5.41; 95%CI: 2.79–10.50) showed a statistically significant association with hearing loss, indicating that older adults have higher relative risks for hearing loss.

**CONCLUSION:**

In the adjusted analyses controlled for multiple risk factors there was no association between hypertension and hearing loss. The dichotomous variable age (being 60 years or older), on the other hand, has shown a significant association with hearing loss.

## INTRODUCTION

Hypertension is the most frequent among non-transmissible chronic diseases. Hypertension is a circulatory disorder, considered chronic, asymptomatic, and of multifactorial origin, characterized by sustained rise in blood pressure levels^1^.

In Brazil, hypertension affects 36 million adults, and more than 60% of the older adults^2^. It is estimated that about 600 million people worldwide live with hypertension, and a global growth of 60% of the cases is expected by 2025^3^.

The overall prevalence of moderate to severe hearing loss is estimated to increase with age, from 2% at age 20 to 12% at age 60 and to over 58% at age 90, with the vast majority of affected persons living in low and middle-income countries. It is worth mentioning that hearing loss is influenced by several determinants such as genetic factors, chronic diseases, environmental factors (noise and use of ototoxic medications) and lifestyle, and that it may entail several losses at economic, social, and quality of life levels^4^.

Some authors also suggest that subjects with hypertension have a greater hearing impairment than subjects without this comorbidity^5^.

One of the pathogenic mechanisms of hypertension which may be involved in hearing loss is the reduction in capillary blood flow due to increased blood viscosity, which may damage the integrity of oxygen and nutrient transport to the cells, causing tissue hypoxia and consequent hearing loss^6,7^. Hypertension may also cause hearing loss because of ionic changes in cellular potentials^7^.

Many studies have been conducted about the association between hypertension and hearing loss, but results are controversial^5^. Some of these studies have observed the existence of a significant association between them^7,8^, while other studies have not found such association^9^.

It is worth mentioning that the aforementioned studies were generally developed in a shorter period, as they are cross-sectional studies, which limits possible conclusions about aspects that interfere in the development of hearing alterations. Longitudinal studies that approach hearing and its relation with chronic diseases are scarce, but may clarify many controversies regarding the influence of some of these risk factors on hearing loss, since we may identify the incident cases of hearing loss, as well as differences in the incidence rates in different groups based on certain exposures, adjusting these analyses for possible confounders.

The objectives of the present study were thus to compare the incidence of hearing loss among adults stratified by the occurrence of hypertension, and to investigate the association between hypertension and hearing loss.

## METHODS

The *Estudo Longitudinal da Saúde do Adulto* (ELSA-Brazil, Brazilian Longitudinal Study of Adult Health) is a multicenter cohort study consisting of 15,000 employees, and conducted in six higher education and research public institutions in the Northeast, South, and Southeast regions of Brazil. All active or retired employees aged 35 to 74 years, of both genders, were eligible for the study. The design, objectives, and profile of the ELSA-Brazil cohort are detailed and published in a previous study^12^.

The ancillary study *Estudo Longitudinal da Saúde Auditiva do Adulto* (ELSA-A, Longitudinal Study of Adult’s Hearing Health) is part of the ELSA-Brazil research center in São Paulo. It was approved by the Ethics Committee of the institution (n° 883/09), and all participants signed the informed consent form.

The ELSA-Brazil research center in São Paulo has 5,061 participants, among which 901 subjects (wave 1) agreed to audiological monitoring as part of the evaluation, and are part of ELSA-A^13^. As in the main study, participants are followed-up, and return for reevaluations in cycles of 3 to 4 years.

At each wave, volunteers answer a questionnaire about health and other personal details, and undergo a series of evaluations, laboratory tests and physical examinations^12,14^.

Some of the procedures for audiological evaluation are: audiological anamnesis, meatoscopy, measurement of acoustic immitance, pure-tone audiometry, and speech audiometry^13^.

### Outcome

At ground zero (wave 1), we identified participants with no hearing loss (n = 676) among the 901 subjects who participated in ELSA-A. Hearing loss was considered as hearing thresholds above 25 dBHL^15^. The mean hearing thresholds by air conduction in the frequencies from 500 Hz to 8 kHz were calculated for each ear and for both ears.

Of the 676 participants, additional 81 subjects were excluded in wave 2 (n = 595), because there was no data from the audiological evaluation for various reasons (death, dropout from the study, or non-attendance at the evaluation even after several appointments).

### Exposure

Hypertension, considered an exposure variable, was defined based on: report of a medical diagnosis of hypertension; and/or use of medications to treat hypertension; and/or systolic blood pressure ≥ 140 mmHg or diastolic blood pressure ≥ 90 mmHg^12,16^.

### Covariates

Covariates considered risk factors for hearing loss were included and adjusted in the analyses performed in the study: sex, age group (up to 39 years, 40 to 49 years, 50 to 59 years, 60 years or older), education (higher education, high school, elementary school), race/ethnicity (white, non-white), income (< USD 1245, 1245–3319, ≥ 3320), smoking (yes, no, former smoker), occupational noise exposure (yes, no), and diabetes (yes, no). Diabetes was defined by the presence of one of the factors: medical history of diabetes; reported use of medications to treat diabetes; fasting serum glucose ≥ 126 mg / dl; glycated hemoglobin level (HbA1c) ≥ 6.5%, or 2-h oral glucose tolerance test with 75 g glucose ≥ 200 mg / dl)^12^.

### Statistical Analysis

Descriptive analysis was performed by calculating the prevalence of variables in wave 1, and the incidence for the outcome of interest.

Analysis of losses (n = 81) was performed, and when compared to the follow-up subjects (n = 595), the proportions of the variables had no significant variation.

To assess the unadjusted association of exposure with the outcome, crude relative risks were calculated.

Three different models (right ear, left ear, and both ears), using Poisson regression with robust variance, were used to calculate the relative risk estimates with respective confidence intervals (95%) for adjusted models between hearing loss and hypertension. Adjustments were performed with sociodemographic variables, and all analyses were stratified by left ear, right ear, and both ears.

The significance level adopted was 5% (p < 0.05), and analyses were performed on the SPSS software version 21.0.

## RESULTS

Wave 1 included 676 subjects. Most subjects (75.9%) were between 40 and 59 years old, female (56.1%), and self-reported as white (57.5%). Regarding the level of education, 43.9% of the subjects had higher education, and income between USD 1245–3319 (Table 1).

**Table 1:** Socio-demographic and general health characteristics at baseline – Wave 1 (n = 676).

	n	%	p^a^
Age group			
Up to 39 years	86	12.7	< 0.001
40–49 years	324	47.9	
50–59 years	189	28.0	
60 or older	77	11.4	
Gender			
Man	297	43.9	0.020
Woman	379	56.1	
Race/ethnic^b^			
White	389	57.5	< 0.001
Non-white	283	42.1	
Education			
Higher education	297	43.9	< 0.001
High-school	308	45.6	
Fundamental education	71	10.5	
Income^b^			
< USD 1,245	217	32.3	< 0.001
USD 1,245–3,319	310	46.1	
≥ USD 3,320	145	21.6	
Hypertension			
No	474	70.1	< 0.001
Yes	202	29.9	
Diabetes			
No	586	86.7	< 0.001
Yes	90	13.3	
Use of hypertension medicine			
No	535	79.1	< 0.001
Yes	141	20.9	
Smoking			
No	387	57.2	< 0.001
Yes	96	14.2	
Former smoker	193	28.6	

^a^ Chi-square test.

^b^ Variables with missing values.

USD – US dollars.

As for general health characteristics, approximately 30% of the subjects had hypertension, and 13.3% had diabetes. Regarding smoking, 57.2% of the subjects had never smoked (Table 1). In wave 2, 35% of the subjects had hypertension.

As for hearing health, in waves 1 and 2 most subjects did not have hearing complaints (78.1% and 64.6%, respectively). As for tinnitus, 36.7% of the subjects in wave 1, and 24.1% in wave 2 reported this symptom, and more than one third of the subjects reported noise exposure in both waves (39.1% and 35.1%, respectively) (Table 2).

**Table 2:** Hearing health characteristics in waves 1 and 2 (n = 676 – Wave 1; n = 595 – Wave 2).

	Wave 1		Wave 2	

	n	%	n	%
Hearing complaint^b^				
No	524	78.1	379	64.6
Yes	147	21.9	208	35.4
p^a^	< 0.001		< 0.001	
Tinnitus^b^				
No	427	63.2	451	75.9
Yes	248	36.7	143	24.1
p^a^	< 0.001		< 0.001	
Exposure to noise^b^				
No	377	60.9	385	64.9
Yes	242	39.1	208	35.1
p^a^	< 0.001		< 0.001	

^a^ Chi-square test.

^b^ Variables with missing values.

In the crude analysis for both ears the incidence of hearing loss was 6.7%, being higher for subjects with hypertension (9.7% versus 5.4%). The relative risk for hearing loss was almost double for subjects with hypertension for the right ear (1.93; 95%CI: 1.10–3.39), and there was a trend toward statistical significance for both ears (Table 3).

**Table 3:** Incidence of hearing loss and estimate on risk for hearing loss with exposure for hypertension.

	Incidence of hearing loss (n = 595) n (%)	Incidence of hearing loss hypertension (n = 174) n (%)	Incidence of hearing loss no-hypertension (n = 421) n (%)	Relative risk (95%CI)	p^a^
LE	55 (9.2)	20 (11.4)	35 (8.3)	1,383 (0.822–2,326)	0.160
RE	45 (7.6)	20 (11.4)	25 (5.9)	1,936 (1,105–3,391)	0.016
BE	40 (6.7)	17 (9.7)	23 (5.4)	1,780 (0.98–3.26)	0.080

^a^ Chi-square test; LE – left ear; RE – right ear; BE – both ears.

In the multivariable model adjusted by the covariates age group, gender, education, income, race, hypertension, diabetes, smoking, and noise exposure, the dichotomous variable age (60 years or older) showed a statistically significant association with hearing loss (for the right ear, left ear, and both ears), indicating that the older adults have a higher relative risk for hearing loss (RR = 5.4; 95%CI: 2.79–10.50). After adjusting for covariates, there was no association between hypertension and hearing loss for any of the analyses (Table 4).

**Table 4:** Association for hearing loss on right ear, left ear and both ears (model adjusted by the covariables age group, sex, education, income, race, hypertension, diabetes, smoking, and exposure to noise).

Variable (ref)	Right ear		Left ear		Both ears	

	RR _adjusted_ (95%CI)^b^	P^ab^	RR _adjusted_ (95%CI)^b^	P^ab^	RR _adjusted_ (95%CI)^b^	P^ab^
Hypertension (no)	1,498 (0.842–2,665)	0.169	1,391 (0.706–2,742)	0.340	1,423 (0.758–2,674)	0.272
Older adults (no)	3,948 (2,105–7,404)	< 0.001	1,390 (0.740–2,611)	0.305	5,415 (2,791–10,508)	< 0.001
Sex (Female)	1,023 (0.556–1,881)	0.942	1,391 (0.706–2,742)	0.340	1,067 (0.563–2,022)	0.842
Education (higher)						
Fundamental	1,395 (0.441–4,413)	0.571	2,647 (1,012–6,919)	0.047	1,507 (0.471–4,819)	0.489
High–school	2,152 (1,072–4,321)	0.031	1,860 (0.947– 3,653)	0.071	1,885 (0.919–3,867)	0.084
Income (Equal to or higher than USD3320)						
Lower than USD 1,245	0.701 (0.301–1,633)	0.410	0.691 (0.258–1,851)	0.462	0.766 (0.301–1,950)	0.577
Between 1,245–3,319	0.318 (0.133–0.756)	0.010	0.760 (0.338–1,711)	0.508	0.431 (0.181–1,026)	0.057
Ethnics/race (White)	0.912 (0.498–1,669)	0.765	0.849 (0.509–1,416)	0.531	0.709 (0.355–1,417)	0.330
Smoking (no)						
Smoker	1,264 (0.536–2,982)	0.592	1,607 (0.772–3,343)	0.204	1,516 (0.583–3,946)	0.394
Former smoker	1,025 (0.530–1,984)	0.941	1,258 (0.738–2,145)	0.399	1,089 (0.555–2,134)	0.804
Diabetes (no)	1,391 (0.706–2,742)	0.340	0.769 (0.376–1,575)	0.473	0.985 (0.433–2,239)	0.971
Exposure to noise (no)	1,390 (0.740–2,611)	0.305	0.974 (0.544–1,744)	0.710	1,751 (0.758–2,674)	0.272

^a^ Chi-square test

^b^ Model adjusted to the variables age group, sex, income (USD-dollar), race, hypertension (hypertension), diabetes, smoking and exposure to noise.

RR = relative risk. Ref – reference category.

## DISCUSSION

In the present study we sought to compare the incidence of hearing loss among adults with and without hypertension, and to check for an association between hypertension and hearing loss. The results of the four-year follow-up of ELSA-A subjects showed an incidence of hearing loss of approximately 7%. There was no association between hypertension and hearing loss after analyses adjusted for multiple risk factors recognized in literature. Older subjects (aged 60 years or more) showed a higher relative risk of hearing loss.

Sociodemographic characteristics of the population studied showed that most subjects were younger adults, which is an advantage in longitudinal studies. The study of aging processes in younger cohorts may provide valid and important prospective measures of exposures, before damage to systems and organs is installed, and while the etiologic processes are developing^17^.

We also observed a higher number of women participating. A previous study^18^ reported that women typically use health services more than men, as they may be more concerned about their health.

White race was self-reported by 57.5% of the participants, and 43.9% of the subjects had higher education. In both cases, the prevalence is above the national averages^19,20^. A previous study on cardiovascular risk factors highlighted that subjects with higher education tend to be more aware about health, as well as healthier habits, besides having more timely access to health services^21^. Therefore, any considerations about health should take into account the education level observed in the sample.

Regarding hypertension, wave 1 disclosed a prevalence of 29.9%, similar to the prevalence of hypertension described in a meta-analysis based on 10 Brazilian cross-sectional studies, which was 28.7%. However, the prevalence of wave 2 (35.8%) was higher than that same study^22^. The higher prevalence of hypertension in wave 2, when compared to wave 1, probably may be explained by the increased age of participants between both waves, which may be a consequence of the aging process^23^.

Regarding hearing health, less than half of the participants reported hearing complaints, tinnitus and exposure to noise, both in wave 1 (21.9%; 36.7% and 39.1%, respectively), and in wave 2 (35.4%; 24.1% and 35.1%, respectively). It is observed an increase in the prevalence of hearing complaints in wave 2, what did not occur with tinnitus and noise exposure.

The increase in the occurrence of hearing complaints in wave 2 may be due to countless factors such as, for example, increase in the number of people with hypertension, aging of participants, and incidence of hearing loss between the two waves. Aging brings with it an increase in chronic diseases, being hypertension and diabetes the diseases with the highest prevalence and incidence in the world^24^.

Studies have reported that these chronic diseases that affect subjects in aging may be related to hearing alterations stemming from age^7,8^.

Moreover, aging *per se* causes a decrease in hearing thresholds, as well as impairment on central auditory processing. Thus, peripheral and central auditory alterations associated with age-related hearing loss may have a negative impact on the perception of verbal and non-verbal auditory stimuli^25^, which could cause an increase in hearing complaints in the second wave.

As for tinnitus, it is known that this symptom often co-exists with hearing loss; however, tinnitus may also occur in people with normal hearing, and it may be an alert for the start of a possible hearing alteration^26^. In wave 1, the 36.7% prevalence of tinnitus even in the absence of hearing loss supports this statement. As for wave 2, a possible explanation for the reduced prevalence of tinnitus complaint, despite the existence of hearing loss in some subjects, is that some of them who had this symptom in wave 1 may not have participated in wave 2 or, in some cases, there may have been remission of the symptom^27^.

More than one third of the subjects who participated in waves 1 and 2 reported exposure to noise. This is a known potential risk for hearing loss and tinnitus^28^. For this reason, noise exposure was included as an adjustment covariate in the analyses performed in this study.

In the crude analysis, the incidence of hearing loss was higher for subjects with hypertension when compared to subjects without hypertension. These findings suggest an increased RR of hearing loss associated with hypertension, in line with previous studies that found that people with hypertension have a higher risk for developing hearing loss^5,29^. However, it should be stressed that this analysis disregarded the effect of covariates.

When investigating hearing loss in subjects with and without hypertension, it is necessary to consider the covariates, trying to exclude their effect on the outcome one intends to study. Thus, in the joint analysis with the possible confounders (age range, sex, education, income, race, hypertension, diabetes, smoking, and exposure to noise), there was no association between hypertension and hearing loss. These findings corroborate the studies by Lin et al.^30^ and Samelli et al.^11^ who did not observe an association between hypertension and hearing loss.

However, still for the adjusted analysis, there was a significant association between being 60 years or older and hearing loss (for the right ear, left ear, and both ears), suggesting that the older adults have an increased RR for developing hearing loss. These results suggest that age is the most important risk factor for hearing loss^5^, since it is the only variable that remained associated with hearing loss after joint analysis with the other confounders.

There is a consensus that age is a major contributor to hearing loss. Age-related hearing loss (or presbycusis) is a symmetrical sensorineural hearing loss that affects especially the higher frequencies, caused by the physiological aging of the cochlea, and the audiometric configuration of age-related hearing loss is similar to that of hypertension^5^. Several studies have shown the influence of age on hearing thresholds^5,29,30^.

In fact, both chronic diseases and hearing loss increase with age, and all these conditions have an impact on the blood microcirculation of the cochlea, which may lead to hearing loss hardly associated specifically with a single risk factor. It is believed that physiological aging would cause hearing loss, and that chronic diseases, such as hypertension, may exacerbate this process^5^.

It should be stressed that variability in literature findings regarding the association between hypertension and hearing loss, including our study, may also be influenced by methodological aspects such as the measures used (self-reported versus objective), criteria adopted (mean used to calculate hearing loss, degrees of hearing loss, etc.), populations assessed (age, race, socio-economic and educational level), the study design (cross-sectional versus longitudinal), as well as covariates incorporated into the analyses^29,30^. For example, Reed et al.^31^ observed no association between hypertension and hearing loss in a cross-sectional analysis, but found an association between hypertension in middle age and hearing loss measured 25 years later, suggesting that the duration of illness may be another influencing factor. Lin et al.^30^ suggested that this inconsistency in the results of the various studies may be related to a weak association between cardiovascular risk factors with hearing loss. Therefore, effects would be masked by stronger risk factors such as age, especially in cohorts with older subjects.

The strengths of this study include: the longitudinal design, the systematic and objective methods used for hearing assessment, laboratory tests, and physical examination of subjects. However, one of the limitations of the study was the use of a self-reported variable for the disease duration, when diagnosed prior to the beginning of the ELSA-Brazil study. In the future, it is important to analyze data regarding the disease duration, checking the influence of time of the disease onset on the worsening of hearing thresholds, investigating whether subjects with longer time of diagnosis have greater hearing impairment.

We emphasize that, despite the many controversies about the influence of hypertension on hearing, patients with this condition should be monitored because they are part of a population considered to be at risk for hearing loss. A better understanding of the relationship between hypertension and hearing loss, as well as the influence of other potentially modifiable risk factors, has important clinical implications, since early referral of adults with hypertension (or other chronic diseases) for audiological evaluation may become one of the first strategies to prevent hearing loss.

In brief, the adjusted analyses controlled for multiple risk factors showed no association between hypertension and hearing loss. The dichotomous variable age (being 60 years or older) showed a significant association with hearing loss.
